# 

**Published:** 1896-08

**Authors:** 


### ASSOCIATION OF VETERINARY FACULTIES OF NORTH AMERICA.

THE third annual meeting will be held at Buffalo, New York, September 4, 1896. The headquarters of the Association will be the Genesee House, where a special parlor is provided for the meeting.

The programme consists of the reports of several committees, notably those of the committee on a uniform veterinary degree, and the committee to devise legislation in regard to a uniform examination by State Boards of Veterinary Examiners. The action upon these reports by the Association will be of great interest and importance. The subjects laid over from the last meeting for want of time will now find consideration. These subjects are:

1. A uniform course of instruction, *i. e.*, the subjects that should be taught in every veterinary school and the time devoted to each. Speaker, Prof. J. W. Adams, Philadelphia.
2. Mutual recognition of students by colleges belonging to the Association of Faculties. Speaker, Prof. H. D. Gill, New York.
3. Competitive examination for position in the Veterinary Faculties.
4. College fees.

It is to the interest of the University Veterinary Departments and to the individual veterinary colleges to send representatives to the coming meeting. All voting delegates must be properly accredited by their college authorities.

OLOF SCHWARZKOPF,
Chairman Executive Committee, Association of Veterinary Faculties.

### NEW YORK STATE VETERINARY MEDICAL ASSOCIATION.

The seventh annual meeting of the New York State Veterinary Medical Society will convene at the Genesee House, in the city of Buffalo, on September 4th and 5th, next. The officers of the Society earnestly desire as large attendance at this meeting as possible. The occasion will be full of interest, both professionally and socially, and one that will not again present itself for a number of years, as the United States Veterinary Medical Association meets during the same week in that city, giving us all an opportunity to make acquaintances from all parts of the country, which will be profitable and pleasant. The Genesee House gives special rates to veterinarians, and the trunk-line roads offer 1⅓ rates.

Claude D. Morris,
Secretary.

Missouri State Veterinary Medical Association meets at Mexico, Mo., on August 26, 1896. Papers on the following subjects will be presented: "The Veterinary Profession;" "Impurities of St. Louis Dairies;" "Parasites Common to the Ox and Sheep;" "Leucocythæmia (With Report of Case in a Dog);" "Remarks on Texas Fever;" "The County Practitioner," and Reports of Cases.

The semi-annual meeting of the Pennsylvania State Veterinary Medical Association will be held in October this year at Reading, Pa. This change from September to October has been made owing to the many important State meetings and the United States meeting occurring about the regularly appointed time. An excellent programme is announced, and will appear in the September issue.

### PERSONAL.

Veterinarian E. W. Heggie, of Lexington, Ky., was one of the judges of thoroughbreds at the recent horse-show at Toronto.

Dr. Leonard Pearson, State Veterinarian, retired from practice on July 1st, owing to the great demands on his time in his official position.

Dr. J. E. Ryder was among the unfortunates at the recent fire in the American Horse Exchange, New York City, having lost part of his office equipment.

Veterinarian Hirsch has invented a rubber shoe for horses that covers the entire foot and laces up the front at the coronet. It is intended to be removed from the feet when in the stable.

Dr. Bartholomew, of Berwyn, was an interested visitor at the Devon open-air horse-show.

One of Pennsylvania's coterie of M.R.C.V.S.'s languishes in one of the jails adjacent to Philadelphia.

Dr. George S. Baker, Inspector of the Bureau of Animal Industry, has been transferred from Chicago to San Francisco, where he is in charge of that port.

Dr. J. H. Adamson, of Washington, D. C., has been sojourning for a period in the region of St. Paul, Minn., in quest of better health.

Prof. Williams, of Bozeman, has been on a three weeks' vacation for rest and recuperation. Out of hearing of locomotives, away from telegraph-lines and an uncertain weekly mail, one feels like envying him under such pleasant circumstances.

Prof. R. S. Huidekoper, editor of the JOURNAL, has gone to the White Mountains with the hope that the higher altitude may more rapidly restore his impaired health, which we are sure every JOURNAL reader wishes for him.

Dr. James B. Rayner, of West Chester, has been quite seriously ill, but we are glad to report his convalescence.

Dr. A. J. Sheldon, of Springfield, Mass., has removed to Boston, and may be addressed at 50 Village Street.

Dr. E. P. Schaffter, of Mt. Eton, Ohio, has accepted an appointment in the Bureau of Animal Industry, and has been stationed at Kansas City.

A. S. Alexander, V.S., of Evanston, Ill., has been appointed an Assistant State Veterinarian. He is also instructor of Hygiene, Breeding and Management of Domesticated Animals in the Chicago Veterinary College, veterinary editor of the *Breeders' Gazette*, and proprietor of the remedy, Umbilicure, sold in the markets.

Dr. T. J. Turner, formerly State Veterinarian of Missouri, successfully passed the civil-service examination, and has been appointed an inspector and assigned for duty at Kansas City, Missouri.

State Veterinarian White, of Missouri, was denied the right at St. Louis of testing the cows for tuberculosis under the recent act governing the milk-supply of that city.

Dr. S. J. J. Harger has an interesting article in the *Whip and Spur* of July 16th, on "Milk of the Buffalo."

Dr. W. A. Porter, of Dunksburg, Mo., has succeeded to the practice of the late Dr. W. E. Smith, of Sedalia, Mo.

Dr. Alexander Glass and family, of Philadelphia, are quartered for the summer at Chelsea, on the Atlantic coast.

Dr. H. D. Gill, of New York City, has recently taken to agricultural pursuits as a side-issue in the scope of his many lines of work.

Dr. C. A. Cary, of Alabama, is travelling through the West, and has been a recent visitor to the Chicago, McKillip, and Kansas City Veterinary Colleges.

Dr. Joseph Hughes, of Chicago, has been on a trip from his city in search of rest and recreation.

Dr. J. P. Turner, Fort Meyer, Va., has been on a leave of absence for fifteen days at his home in West Chester and among his professional friends in Philadelphia and vicinity.

Dr. Charles H. Ford, late House Surgeon of the United States College of Veterinary Surgeons at Washington, D. C., has gone to locate in practice at New Orleans, La.

Dr. Fred. Torrance, of Brandon, Manitoba, holds the position of district veterinarian for his locality.

Dr. C. P. Lyman, of Boston, has repaired to his farm at Jaffrey, N. H., for a period of rest.

At both examinations conducted by the Pennsylvania State Board all the members, namely, Messrs. Harger, Hoskins, McNeil, Sallade, and Walters, have been present to conduct the examinations.

Prof. Hermann M. Biggs, Director of the Bacteriological Laboratories of the Health Department, City of New York, sailed for Europe July 1st, seeking recreation and at the same time visiting the various bacteriological laboratories of Europe.

Dr. Thomas Giffin, with his family, will spend the summer at Long Branch.

Dr. H. D. Gill has observed two cases of localized tetanus.

Dr. M. O'M. Knott is located at Plainfield, N. J., where we learn he is enjoying the confidence of the people and reaping the benefit thereof.

Dr. Henry Henning, for a number of years assistant of the late Dr. James Hamill, of New York, is now located at 512 East Seventeenth Street, where he has opened a hospital.

Prof. Essling, of Berlin, has been sent to this country by the German courts to investigate the Kneeb case. The latter still lingers in confinement, and the mare Bethel, said to be a ringer, is now on the Kneeb farm in Nebraska. She will be examined, and other information sought concerning this now famous case.

We trust that Prof. Essling may be a visitor to our U. S. V. M. A. meeting at Buffalo in September.

Veterinarian C. W. Stowe, of Saginaw, Mich., has taken an active interest in the Master and Journeymen's Horseshoers' School conducted there during the past winter.

